# 80 Improving policy and practice for cyclists with a disability in The Netherlands

**DOI:** 10.1093/eurpub/ckae114.081

**Published:** 2024-09-26

**Authors:** Robin Rauws, Mark Noordzij, Caroline van Lindert

**Affiliations:** Mulier Instituut, Utrecht, The Netherlands; Mulier Instituut, Utrecht, The Netherlands; Mulier Instituut, Utrecht, The Netherlands

## Abstract

**Purpose:**

Cycling is known to have great health benefits and to contribute to the autonomy and independence in mobility of people with a disability. However, Dutch streets and cycling lanes are not designed for adapted bicycles, and other traffic users often do not know how to react to cyclists with an adapted bicycle. Furthermore, there is a lack of cycling policy that accounts for disabled cyclists. This raises the following questions:

- Why, when, and where do people with a disability cycle?

- What challenges do cyclists with a disability experience when cycling?

- How can these challenges be addressed, according to people with a disability themselves and professionals in the field?

This study will help to gain insight into the practice of cycling among Dutch adults with a disability, in order to better understand their challenges and needs. A better understanding of these challenges and needs is necessary to make cycling more accessible. We also aim to raise awareness amongst designers and policy officials of the great diversity within cyclists’ needs and the types of bicycles used. We ultimately hope to contribute to greater cycling participation among people with a disability.

**Methods:**

We conducted ten interviews with Dutch adults with different disabilities who shared their experiences of cycling in their neighborhood environment and beyond. The interview transcripts were coded and analyzed based on main themes we identified: characteristics of general cycling behavior, motivation(s) to cycle, challenges in cycling and possibilities to improve the cycling experience. We discussed these results for improvement in a workshop with professionals in spatial and traffic design.

**Results:**

We will present the experiences of cyclists with a disability, the challenges they face and suggestions for improvements.

**Conclusions:**

With this study, we hope to contribute to a better understanding of the needs and experiences of cyclists with a disability. Applying these insights will help policy officers and other professionals in the field in making their policies and designs more effective and improve road designs for cyclists with a disability.

**Funding:**

This research project is funded by the Dutch Ministry of Health, Welfare and Sport (VWS).

